# Pharmacovigilance Insights into Ibuprofen’s Neuropsychiatric Safety: A Retrospective Analysis of EudraVigilance Reports

**DOI:** 10.3390/ph18091301

**Published:** 2025-08-29

**Authors:** Cristina Anamaria Buciuman, Carmen Maximiliana Dobrea, Anca Butuca, Adina Frum, Felicia Gabriela Gligor, Mihai O. Botea, Laura Grațiela Vicaș, Mariana Eugenia Mureșan, Octavia Gligor, Florin Maghiar, Alexia Manole, Claudiu Morgovan

**Affiliations:** 1 Faculty of Pharmacy, Carol Davila University of Medicine and Pharmacy, 6 Traian Vuia Str., 020956 Bucharest, Romania; buciuman.apetrii@drd.umfcd.ro (C.A.B.); lvicas@uoradea.ro (L.G.V.); 2 Preclinical Department, Faculty of Medicine, “Lucian Blaga” University of Sibiu, 550169 Sibiu, Romania; carmen.dobrea@ulbsibiu.ro (C.M.D.); anca.butuca@ulbsibiu.ro (A.B.); adina.frum@ulbsibiu.ro (A.F.); felicia.gligor@ulbsibiu.ro (F.G.G.); claudiu.morgovan@ulbsibiu.ro (C.M.); 3 Faculty of Medicine and Pharmacy, University of Oradea, 10, 1 December Square, 410073 Oradea, Romania; mbotea@uoradea.ro (M.O.B.); mmuresan@uoradea.ro (M.E.M.); fmaghiar@uoradea.ro (F.M.); manole.alexia@student.uoradea.ro (A.M.)

**Keywords:** pharmacovigilance, neuropsychiatric safety, ibuprofen, neuropsychiatric disorders, NSAIDs

## Abstract

**Background/Objectives**: Mental health awareness is rising; thus, neurological and psychiatric side effects also benefit from increased attention from the medical and scientific community. Ibuprofen is a well-known non-steroidal anti-inflammatory drug (NSAID) that is often available over-the counter (OTC) for both adults and children, expressing good efficacy in reducing pain and fever through non-selective cyclooxygenase inhibition. As ibuprofen has already been associated with different neuropsychiatric disorders, the aim of this study was to perform an up-to-date analysis of such signals detected in the cases reported in EudraVigilance (EV). **Methods:** The disproportionality analysis offered a contextual insight into the real-world situation depicted in the analyzed database. **Results**: From the total cases reported for ibuprofen (*n* = 58,911), 13.9% contained nervous system disorders (*n* = 8214) and 10.7% entailed psychiatric disorders (*n* = 6295). The cases were distributed between all age groups, with a sensible higher incidence in teenagers and in women in general. Severe cases, including deaths, have been reported. By comparison with ketoprofen, acetylsalicylic acid, and diclofenac, ibuprofen presented a higher probability of reporting psychiatric and behavioral symptoms. Regarding cognitive and attention disorders and disturbances, no disproportionate signal was observed between ibuprofen and all other NSAIDs. Sleep disturbances (hypersomnia, narcolepsy and sleep paralysis) are reported as more probable for ibuprofen than for acetylsalicylic acid, naproxen, and diclofenac. A higher risk of reporting suicidal and self-injurious behaviors was noted for ibuprofen versus all other selected NSAIDs. A limitation of the study can be noted as due to suspected causality, not an established one, and EV reports cannot accurately determine adverse drug reaction frequencies. **Conclusions**: Considering that ibuprofen is easily accessible as an OTC drug and the higher probability of reporting several neuropsychiatric adverse effects as shown by this study, patient counseling, when possible, and general education for the public are valuable tools in managing these adverse reactions.

## 1. Introduction

Ibuprofen is one of the most demanded nonsteroidal anti-inflammatory drugs (NSAIDs), analgesics, and antipyretics, with increasing popularity since its approval in Europe in 1969 and in the United States of America in 1974 [1,2]. This position could be reached and maintained due to a series of concurrent key factors, which contribute to a positive outcome for the main healthcare stakeholders: patients, healthcare specialists, and the health system [3,4,5]. A multitude of pharmaceutical dosage forms of ibuprofen are available over the counter (OTC) or as prescription drugs, to optimize the administration of active pharmaceutical ingredients through various routes (oral [6], dermal [7], rectal [8], intravenous [9]), both for adults and children [10].

Ibuprofen inhibits cyclooxygenase (COX) enzymes, blocking the transformation of arachidonic acid mediated this way. Three isoforms have been described: a constitutive one (COX-1), an inducible one (COX-2) and a third form which needs further understanding (COX-3). Ibuprofen non-selectively inhibits both COX-1 and COX-2, with greater affinity for COX-1 (2.5-fold), accounting for its analgesic, antipyretic, and anti-inflammatory properties [11].

Despite their common mechanism of action, the safety profile of NSAIDs could be different. Ibuprofen is generally well tolerated, but certain categories of adverse effects were repeatedly reported over the years and have been stated also on the leaflet: gastrointestinal events (abdominal pain, dyspepsia, bleeding, etc.) [12], cardiovascular effects (new-onset or exacerbation of heart failure [13], venous thromboembolism [14], hypertension [15], myocardial infarction [16], etc.), liver injury [17], hypersensitivity reactions [18], kidney injury [19], etc.

Neurological and psychiatric side effects have benefited from increased attention from the medical and scientific community, as mental health awareness is rising. Neuropsychiatric adverse reactions (ADRs) to several drugs can affect the daily functioning and quality of life of patients, and their ability to adhere to prescribed medication regimens; thus, these ADRs can lead to non-compliance, treatment discontinuation, or disease relapse [20]. Associated with ibuprofen-use, different neuropsychiatric disorders have been reported: aseptic meningitis [21], altered consciousness [22], ataxia, dizziness, headache [23], sleep disorders [24], depression [25], etc.

The exact mechanisms responsible for the neurological and psychiatric side effects of ibuprofen are not fully understood but based on current knowledge, several theories have been proposed: (i) the inhibition of prostaglandin in the central nervous system; (ii) the penetration of the blood–brain barrier; (iii) the altered activity of neurotransmitters; (iv) hypersensitivity or idiosyncratic reactions [26,27,28,29,30]. Further research is needed to elucidate these mechanisms fully.

Pharmacovigilance studies enable the continuous evaluation of medicines, after they obtain marketing authorization. Authorities like the World Health Organization, the European Union (EU), and the Food and Drug Administration of the United States of America (FDA) encourage the spontaneous reporting of adverse events, then collect and assess the reports and have developed dedicated databases to facilitate the complex analysis of the curated data. In pharmacovigilance, the most common method for collecting data is passive spontaneous reporting. This represents a voluntary reporting of ADRs by health or non-health (patients) professionals. Although active reporting from specific studies (e.g., large-scale post-marketing surveillance studies, patient registries, phase IV clinical trials, etc.) provides more controlled and systematic data, the spontaneous method can gather a wide range of real-world data over a long period of time, offering insights into rare, serious, and long-term adverse effects. However, after the ADR’s reporting, additional investigations to determine a causal relationship between the drug and the occurrence of the ADR should be established (the evaluation of the likelihood that a particular treatment is the cause of an observed adverse event) [31]

EudraVigilance (EV) is a system for collecting reports of suspected ADRs. This information is used to assess the benefit–risk balance in the development phases of medicines and to monitor their safety after authorization in the European Economic Area (EEA). EV includes ADRs associated with medicines that have been reported both before and after authorization. This system facilitates the identification of signals related to suspected ADRs that were previously unknown, as well as the discovery of new information regarding already known ADRs. Moreover, EV ensures standardized data cleaning and quality control, providing a reliable basis for pharmacovigilance studies [32].

According to the European Medicines Agency (EMA), an “aspect of a known link between a medicine and a side effect that requires further investigation” is considered a pharmacovigilance signal [32]. The identified safety signals are evaluated and trigger different responses, depending on seriousness [33,34,35]. An ADR could be considered serious if it resulted in death, or lead to persistent or significant disability or incapacity, or a birth defect; it was life-threatening; or required hospitalization or the prolongation of existing hospitalization [36].

As ibuprofen is one of the most accessible analgesic and antipyretic drugs to people of all ages worldwide, special attention should be given to periodically reassess its safety profile. The aim of this study is to identify real-world reports of neurological and psychiatric suspected ADRs related to ibuprofen and to assess their pharmacovigilance significance through the established complementary methods of descriptive and disproportionality analysis. Since many consumers have gaps in knowledge regarding NSAIDs’ safety, and particularly regarding ibuprofen, such accessibility may represent a significant public health issue. Continuous monitoring of suspected ADRs is essential for assessing drug safety profiles; thus, our study provides an up-to-date overview of neuropsychiatric aspects related to the safety profile of ibuprofen and reinforces the public awareness of ibuprofen’s neuropsychiatric effects.

## 2. Results

### 2.1. Descriptive Analysis

**Figure 1:**
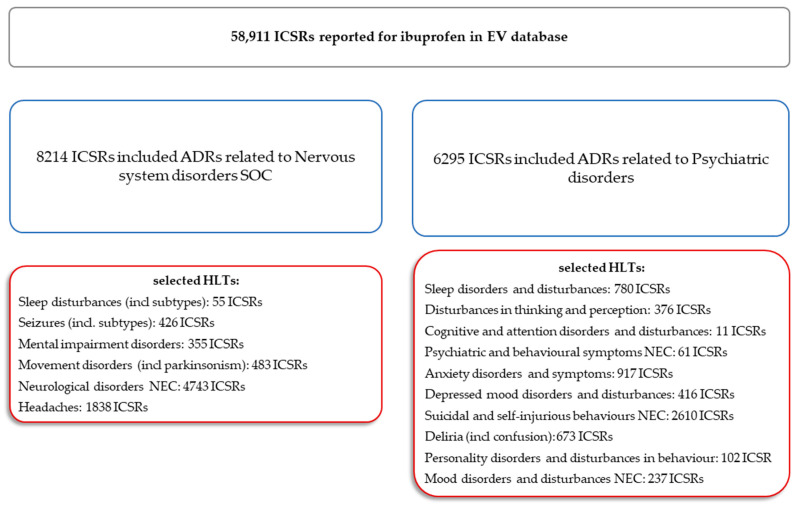
Selection process diagram of Individual Case Safety Reports and their distribution across the System Organ Classes and High-Level Terms of interest. ADR—adverse drug reaction; HLTs—High-Level Terms; ICSR—Individual Case Safety Report; NEC—not classified elsewhere; SOCs—System Organ Classes. Gray—aggregated data for ibuprofen; blue—System Organ Class level; red—High-Level Term level.

From the total cases reported for ibuprofen until 1 June 2025 (*n* = 58,911), 13.9% of total reports contained nervous system disorders (*n* = 8214) and 10.7% contained psychiatric disorders (*n* = 6295). For a total of 16 HLTs from both SOCs (nervous system disorders—6 HLTs, and psychiatric disorders—10 HLTs), a total of 14,083 entries have been found. Neurological disorders not classified elsewhere (NECs) have been included in 4743 reports and headaches were reported in 1838 reports. On the other hand, for psychiatric disorders, the highest number of cases were related to suicidal and self-injury behaviors NEC (*n* = 2610) and anxiety disorders and symptoms (*n* = 917) (Figure 1).

For both categories, the most ADRs have been reported in the 18–64 years group (nervous system disorders, *n* = 4294, 52.9%; psychiatric disorders, *n* = 3476, 55.2%). The next groups most affected by nervous system disorders were the 65–85 years (*n* = 1138, 14.0%) and 12–17 years (*n* = 568, 7.0%) groups. Regarding the frequency of ADRs related to psychiatric disorders, the situation is reversed, with adolescents being more affected (*n* = 751, 11.9%) than 65–85 years old patients (*n* = 556, 8.8%) (Figure S1, Supplementary Materials).

**Figure 2:**
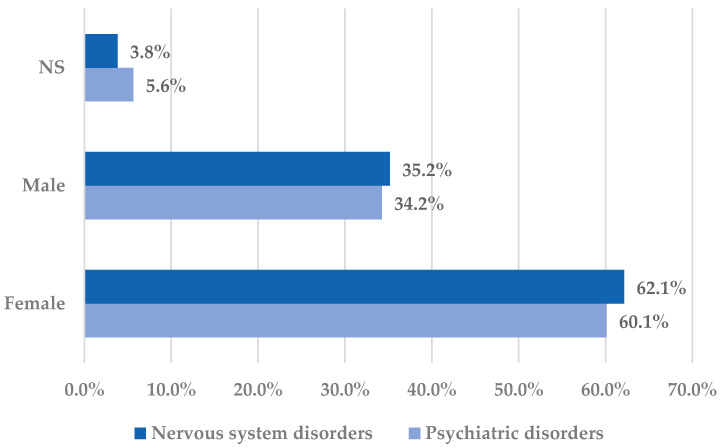
Distribution of cases by sex in the Nervous system disorders and Psychiatric disorders System Organ Classes. NS—not specified.

The distribution of cases by sex in the selected SOCs is presented in Figure 2.

Healthcare professionals are the main reporter category for ADRs from both SOCs, but a higher frequency of the cases related to psychiatric disorders (77.3%, *n* = 5544) than to neurological disorders (68.2%, *n* = 4869) was reported by healthcare professionals (Figure S2, Supplementary Materials).

**Figure 3:**
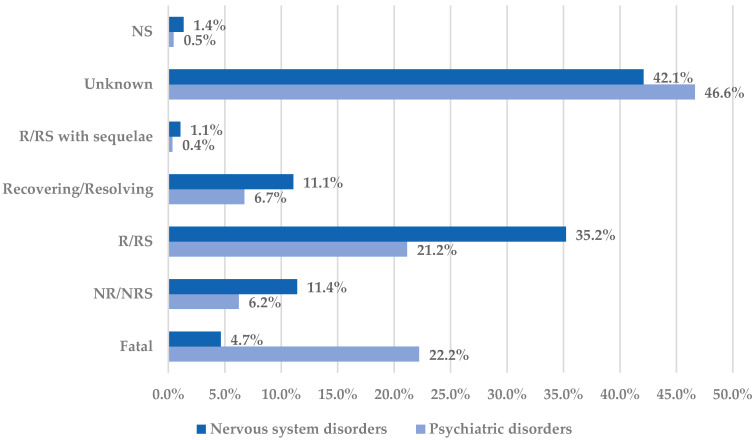
Distribution of cases by outcome in the Nervous system disorders and Psychiatric disorders System Organ Classes. NR—not recovered; NRS—not resolved; NS—not specified; R—recovered; RS—resolved.

A favorable outcome was reported for nervous system disorders (46.8%) more frequently than for psychiatric disorders (27.9%). In contrast, unfavorable outcomes have been reported more frequently in cases associated with psychiatric disorders compared to neurologic disorders (Figure 3).

From neurological diseases, the highest incidence of cases with unfavorable outcomes have been reported for mental impairment disorders (fatal cases—28.5%, and not recovered/not resolved cases—13.0%) and for headaches (fatal cases—6.2%, and not recovered/not resolved cases—17.5%). On the other hand, for psychiatric disorders, the incidence of cases with unfavorable outcomes was the highest for suicidal and self-injurious behaviors NEC (fatal cases—38.6%, and not recovered/not resolved—0.9%), depressed mood disorders and disturbances (fatal cases—13.0%, and not recovered/not resolved—16.3%), sleep disorders and disturbances (fatal cases—10.6%, and not recovered/not resolved cases—17.3%), and cognitive and attention disorders and disturbances (fatal cases—0%, and not recovered/not resolved cases—27.3%). Even with a high incidence of unfavorable outcomes, a not fatal outcome has been reported for cases reporting cognitive and attention disorders. On the other hand, most cases with unfavorable outcome associated with suicidal and self-injurious behaviors were fatal (Figure S3, Supplementary Materials).

### 2.2. Disproportionality Analysis

#### 2.2.1. Psychiatric Disorders

**Figure 4:**
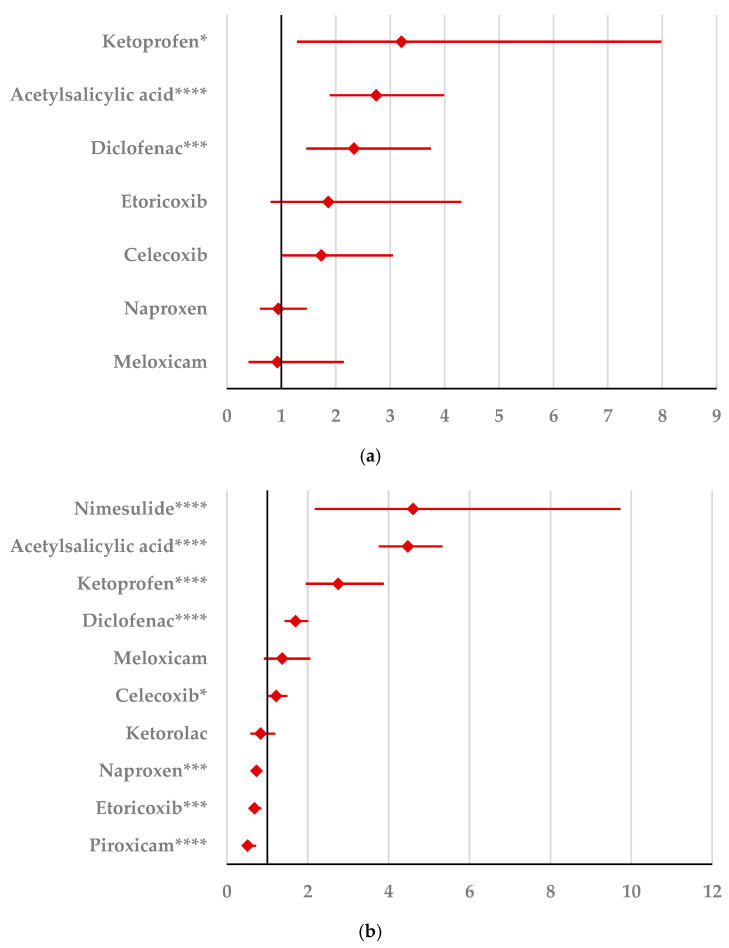

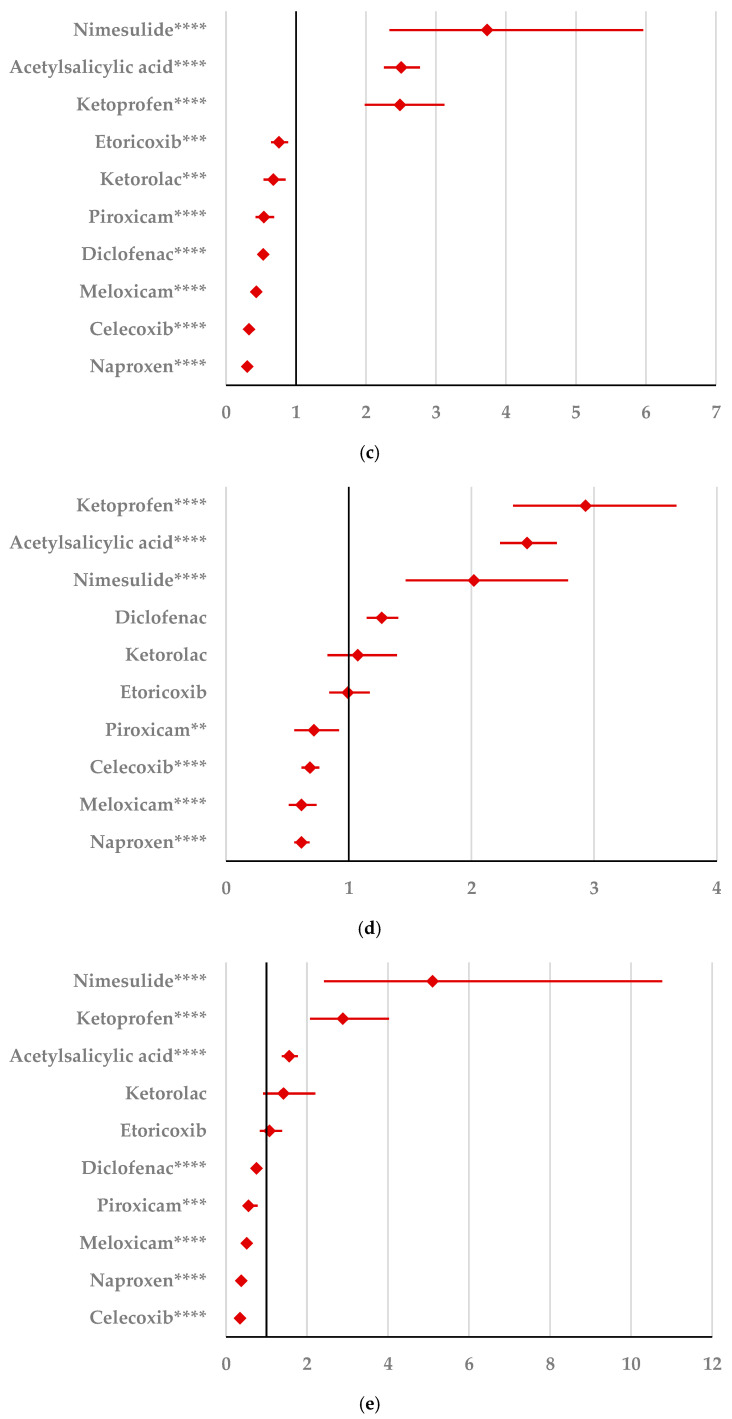

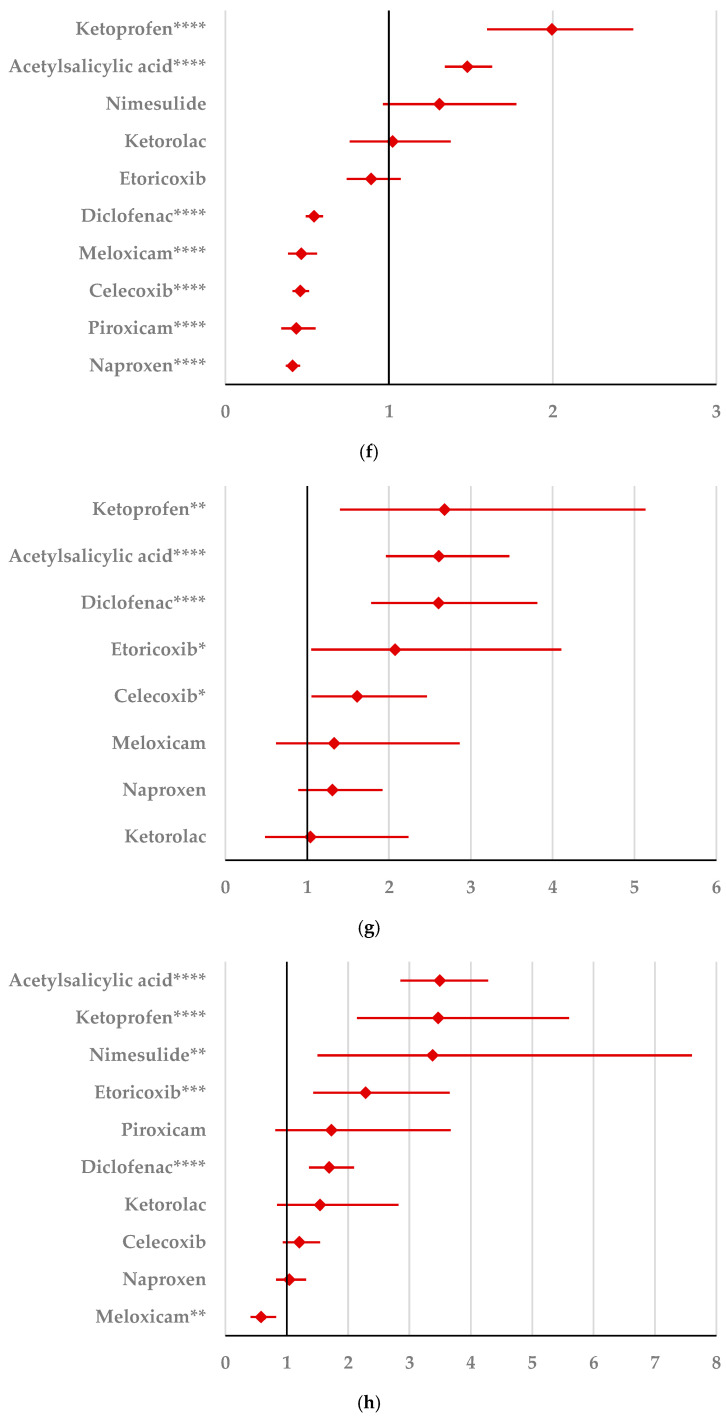

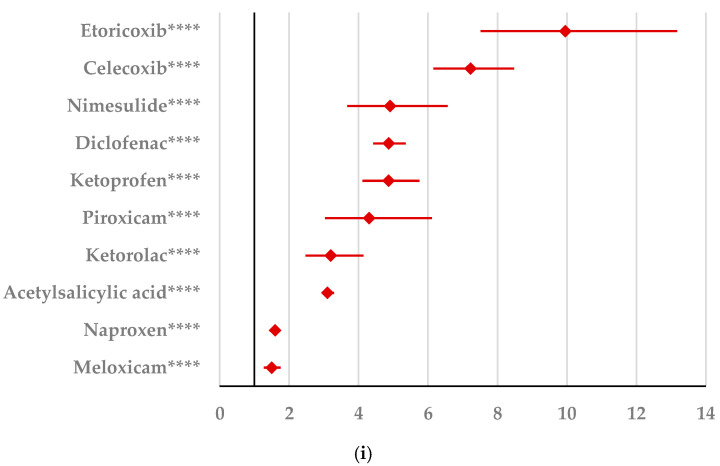
Forest plot for psychiatric disorders: (**a**) psychiatric and behavioral symptoms NEC; (**b**) disturbances in thinking and perception; (**c**) sleep disorders and disturbances; (**d**) anxiety disorders and symptoms; (**e**) depressed mood disorders and disturbances; (**f**) deliria; (**g**) personality disorders and disturbances in behavior; (**h**) mood disorders and disturbances NEC; (**i**) suicidal and self-injurious behaviors NEC. ROR (Reporting Odds Ratio) values are represented on the x-axis, with 95% confidence intervals (CIs) shown. Statistical significance is indicated as follows: * *p* < 0.05; ** *p* ≤ 0.01; *** *p* ≤ 0.001; **** *p* ≤ 0.0001. A higher probability of reporting is considered when the lower bound of a 95% CI is greater than 1.

By comparison with ketoprofen (ROR: 3.21, 95% CI: 1.29–7.98), acetylsalicylic acid (ROR: 2.75, 95% CI: 1.89–3.99), and diclofenac (ROR: 2.34, 95% CI: 1.46–3.75), ibuprofen presented a higher probability of reporting psychiatric and behavioral symptoms NEC (Figure 4a). Also, disturbances in thinking and perception have been reported with a higher probability for ibuprofen than for nimesulide (ROR: 4.60; 95% CI: 2.18–9.73), acetylsalicylic acid (ROR: 4.47; 95% CI: 3.76–5.33), ketoprofen (ROR: 2.75; 95% CI: 1.96–3.88), diclofenac (ROR: 1.70; 95% CI: 1.43–2.01), and celecoxib (ROR: 1.22; 95% CI: 1.00–1.49). In contrast, a low risk of reporting disturbances in thinking and perception could be noticed for ibuprofen in comparison with piroxicam, etoricoxib, and naproxen (Figure 4b). Sleep disorders and disturbances were reported more probably for ibuprofen compared to nimesulide (ROR: 3.73; 95% CI: 2.34–5.96), acetylsalicylic acid (ROR: 2.50; 95% CI: 2.26–2.77), and ketoprofen (ROR: 2.49; 95% CI: 1.98–3.12), and less probable than all other NSAIDs used in the analysis (Figure 4c). Moreover, ibuprofen was associated with a lower risk of reporting anxiety disorders compared to certain NSAIDs (naproxen, meloxicam, celecoxib, and piroxicam), and a higher risk compared to ketoprofen (ROR: 2.93; 95% CI: 2.34–3.67), acetylsalicylic acid (ROR: 2.45; 95% CI: 2.23–2.70), nimesulide (ROR: 2.02; 95% CI: 1.47–2.79), and diclofenac (ROR: 1.27; 95% CI: 1.15–1.40) (Figure 4d). According to data presented in Figure 4e, ibuprofen was associated more probably with depressed mood disorders compared to nimesulide (ROR: 5.10; 95% CI: 2.41–10.77), ketoprofen (ROR: 2.89; 95% CI: 2.07–4.03), and acetylsalicylic acid (ROR: 1.56; 95% CI: 1.38–1.77), and it was less probable in this regard than celecoxib, naproxen, meloxicam, piroxicam, and diclofenac (Figure 4e). Deliria associated with ibuprofen was reported more frequently than for ketoprofen (ROR: 2.00; 95% CI: 1.60–2.49) and acetylsalicylic acid (ROR: 1.48; 95% CI: 1.34–1.63), and less frequently than for naproxen, piroxicam, celecoxib, meloxicam, and diclofenac (Figure 4f). By comparison with ketoprofen (ROR: 2.68; 95% CI: 1.40–5.23), acetylsalicylic acid (ROR: 2.61; 95% CI: 1.96–3.47), diclofenac (ROR: 2.61; 95% CI: 1.78–3.81), etoricoxib (ROR: 2.08; 95% CI: 1.05–4.11), and celecoxib (ROR: 1.61; 95% CI: 1.05–2.46), personality disorders and disturbances in behavior are reported as more probable for ibuprofen (Figure 4g). Mood disorders and disturbances NEC have been reported as more probable than for acetylsalicylic acid (ROR: 3.49; 95% CI: 2.85–4.28), ketoprofen (ROR: 3.47; 95% CI: 2.15–5.60), nimesulide (ROR: 3.38; 95% CI: 1.50–7.60), etoricoxib (ROR: 2.29; 95% CI: 1.43–3.65), and diclofenac (ROR: 1.69; 95% CI: 1.36–2.10). By comparison with meloxicam, these ADRs are reported for ibuprofen with a lower probability (Figure 4h). A very interesting observation could be noticed regarding the higher risk of reporting suicidal and self-injurious behaviors associated with ibuprofen use compared to all other NSAIDs used in the present study (Figure 4i). Regarding cognitive and attention disorders and disturbances, no disproportionate signal was observed between ibuprofen and all other NSAIDs used for comparison (Supplementary Materials, Table S2).

#### 2.2.2. Nervous System Disorders

**Figure 5:**
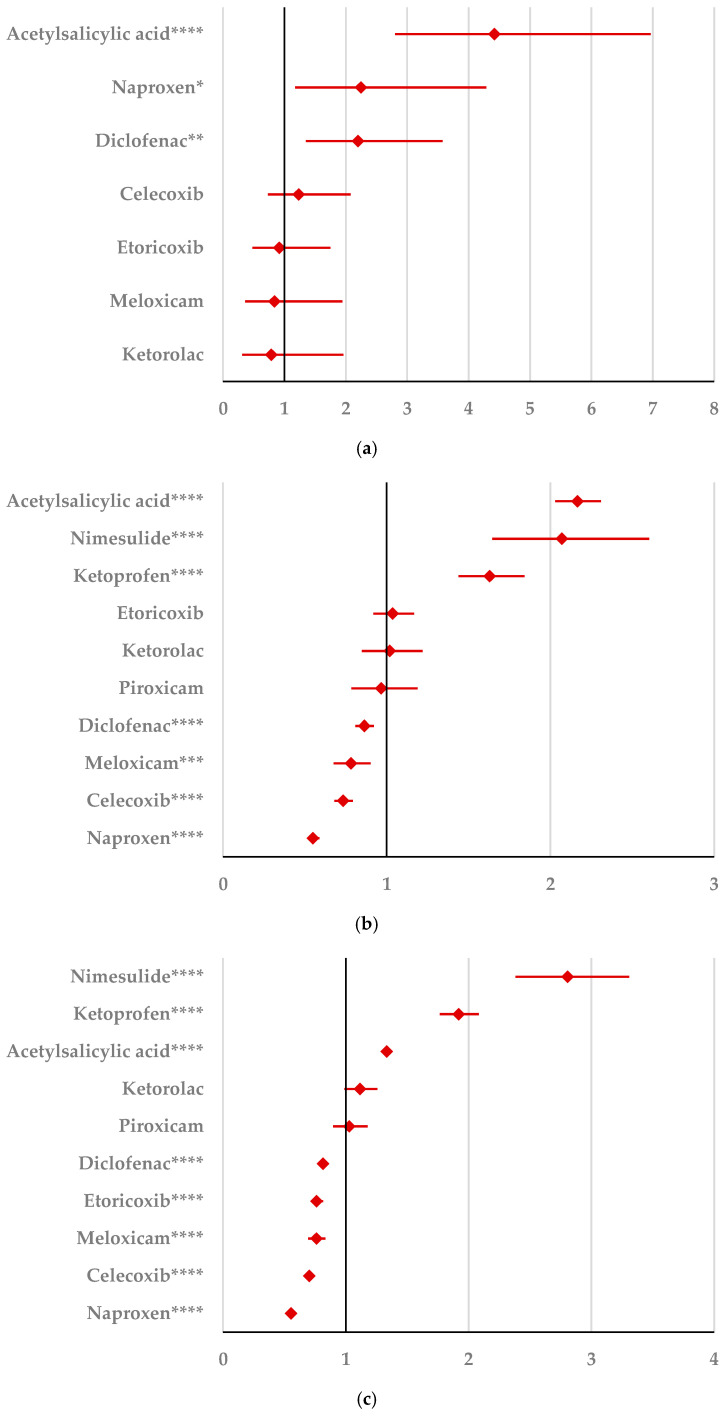

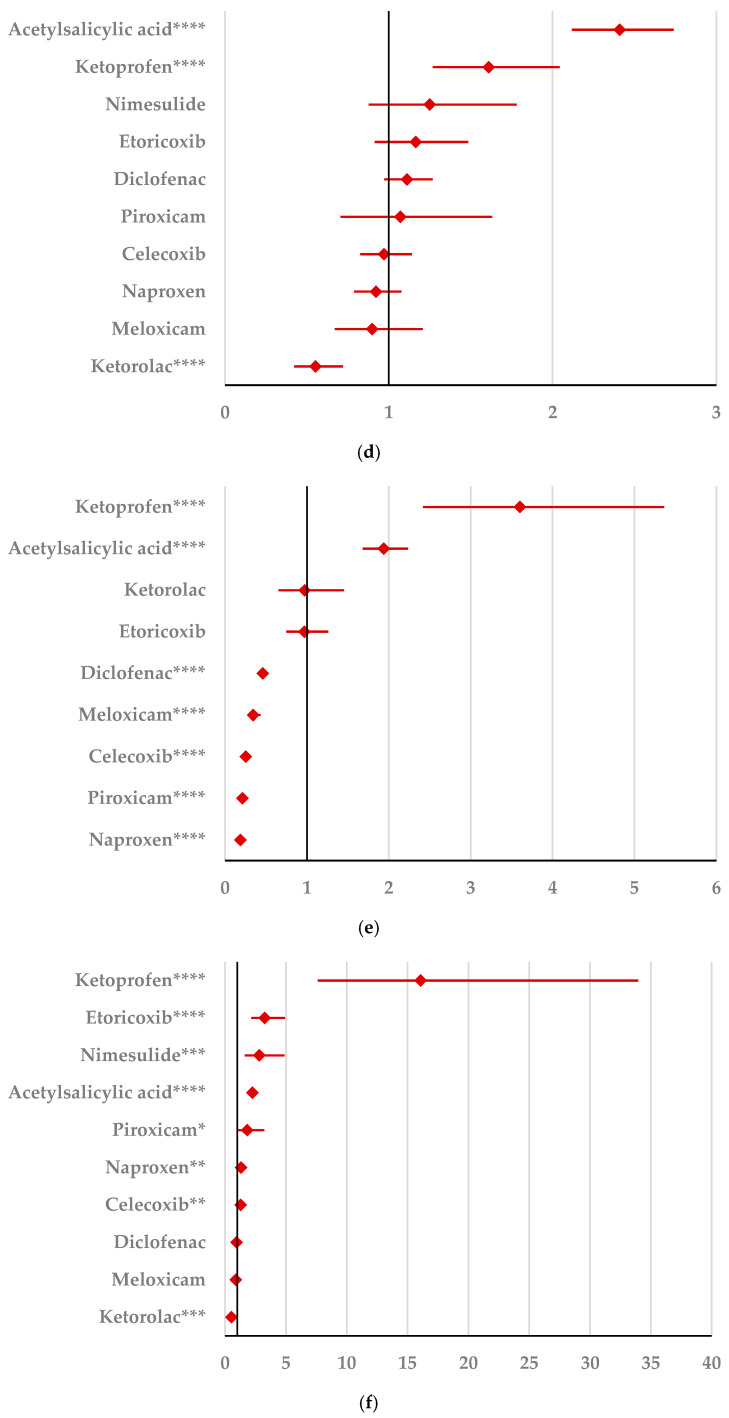
Forest plot for nervous system disorders: (**a**) sleep disturbances (incl subtypes); (**b**) headaches; (**c**) neurological disorders NEC; (**d**) movement disorders (incl parkinsonism); (**e**) mental impairment disorders; (**f**) seizures (incl subtypes). ROR (Reporting Odds Ratio) values are represented on the x-axis, with 95% confidence intervals (CIs) shown. Statistical significance is indicated as follows: * *p* < 0.05; ** *p* ≤ 0.01; *** *p* ≤ 0.001; **** *p* ≤ 0.0001. A higher probability of reporting is considered when the lower bound of a 95% CI is greater than 1.

Sleep disturbances reported as nervous system disorders (hypersomnia, narcolepsy, and sleep paralysis) are reported as more probable for ibuprofen than for acetylsalicylic acid (ROR: 4.42; 95% CI: 2.80–6.97), naproxen (ROR: 2.24; 95% CI: 1.17–4.29), and diclofenac (ROR: 2.20; 95% CI: 1.35–3.58) (Figure 5a). By comparison with acetylsalicylic acid (ROR: 2.17; 95% CI: 2.03–2.31), nimesulide (ROR: 2.07; 9% CI: 1.64–2.60), and ketoprofen (ROR: 1.63; 95% CI: 1.44–1.84), headaches are reported as more probable for ibuprofen, and the opposite, as less probable than for naproxen, celecoxib, meloxicam, and diclofenac (Figure 5b). A similar situation was noticed for neurological disorders NEC (Figure 5c). Thus, these ADRs appear to be more frequent for ibuprofen than for nimesulide (ROR: 2.81; 95% CI: 2.38–3.31), ketoprofen (ROR: 1.92; 95% CI: 1.77–2.08), and acetylsalicylic acid (ROR: 1.33; 95% CI: 1.28–1.38) (Figure 5c). Movement disorders (including parkinsonism) and mental impairment disorders are reported as more probable for ibuprofen than for ketorolac, and as more probable for ibuprofen than for acetylsalicylic acid and ketoprofen. In contrast, ibuprofen had a low risk of reporting movement disorders compared to ketorolac, naproxen, piroxicam, celecoxib, meloxicam, and diclofenac for mental impairment disorders (Figure 5d,e). According to Figure 5f, seizures (including subtypes) seem to be reported as more probable for ibuprofen than for the majority of NSAIDs used in the analysis: ketoprofen (ROR: 16.09; 9% CI: 7.62–33.96), etoricoxib (ROR: 3.27; 95% CI: 2.16–4.93), nimesulide (ROR: 2.81; 95% CI: 1.62–4.88), acetylsalicylic acid (ROR: 2.26; 95% CI: 1.98–2.59), piroxicam (ROR: 1.82; 95% CI: 1.02–3.23), naproxen (ROR: 1.30; 95% CI: 1.08–1.57), and celecoxib (ROR: 1.30; 95% CI: 1.07–1.57).

## 3. Discussions

Pain can directly and negatively influence quality of life [37]; thus patients frequently use NSAIDs as a first-line treatment, with ibuprofen being one of the most accessible. Evaluating the link between genetic, epigenetic, and pharmacological factors and drug interactions is essential for optimizing therapies and understanding adverse reactions that may occur in the process. Due to their mechanism of action, NSAIDs may interfere in certain psychiatric or nervous system disorders by modulating inflammatory pathways and potentially affecting epigenetic mechanisms. Moreover, a deeper understanding of the genetic and epigenetic factors that influence an individual’s response to NSAIDs could facilitate the development of more personalized medicine [38,39]. This study evaluated the neuropsychiatric safety profile of ibuprofen based on spontaneous reports submitted in EV database. Thus, our study revealed that out of the total cases of ADRs reported for ibuprofen, 13.9% contained nervous system disorders and 10.7% contained psychiatric disorders. The neurologic effects of ibuprofen administration have not only been regarded as a safety concern, but also as having potential pharmacological implications—the determination of neuroanatomical changes that need to be further studied. The exact mechanism leading to nervous system function modification still needs to be studied [40].

A previous study attempted to establish the influence of ibuprofen administration on brain age using the BrainAGE model (brain age gap estimation was defined as the difference between kernel-estimated brain age and chronological age). According to Le et al., ibuprofen (placebo, 200 mg, or 600 mg of ibuprofen was administered in a double-blind crossover study), due to its acute anti-inflammatory effects, it temporarily reduces BrainAGE by approximately one year [41]. This effect associated with ibuprofen might represent an opportunity for future studies in order to enhance clinical benefits of ibuprofen.

Regarding age distribution, our study revealed that the highest number of cases have been reported in the 18–64 year age range. For the second most affected group, the situation differs according to the category of ADRs as follows: nervous system disorders were more frequent in the 65–85 years group than in the 12–17 years group and the opposite was shown for psychiatric disorders. Our findings regarding the distribution of most ADRs in the adult group are similar to those of other studies [28,40].

Both for psychiatric disorders and nervous system disorders, females represented the majority of the reported cases (over 60%). Apparently, analgesic effects predominate in men, although this remains unclear since pharmacokinetic discrepancies have not been observed. The alleged sex difference in nociception could be associated with estrogen effects on the activity of the nervous system, thus resulting in an improved transmission of pain impulses [42,43]

Our study showed that approximatively 13% of total reports were represented by headache (among nervous system disorders) and 18% were represented by suicidal and self-injury behaviors NEC (among psychiatric disorders). Headache associated with ibuprofen consumption has been mentioned before in the literature, due to frequent and excessive use of NSAIDs (e.g., ibuprofen or naproxen) for the treatment of migraine, and the most likely cause would be the rebound effect [44,45]. Regarding suicidal and self-injury behaviors associated with ibuprofen, controversial data has been mentioned in the literature. Lehrer et al. conducted a study to establish the idea that NSAIDs reduce suicidal ideation and depression based on the anti-inflammatory effect of ibuprofen [46]. Also, another study supports this supposition [47]. However, an exploratory pharmacovigilance study on the FDA database evaluating the 20 medications that are most common associated with suicidal ideation and self-injurious behaviors exposed ibuprofen as giving notable reporting frequencies of the above mentioned adverse events [48]. Moreover, a case of deliberate ingestion of an overdose of sustained-release ibuprofen was cited in the literature as having a fatal outcome [49]. The scientific literature mentions high dose of ibuprofen involved in serious nervous system disorders, more than 400 mg/kg specifically for pediatric patients, or more than 3200 mg/day. Safe therapeutic doses of ibuprofen are considered: in adults this is up to 1200–2400 mg/day for OTC administration and a maximum of 3200 mg/day under medical supervision; in children this entails a 5–10 mg/kg/dose, not to exceed 30–40 mg/kg/day. Severe adverse reactions, including neurological ones, are mentioned in the literature, especially at doses >400 mg/kg in children or in intentional/accidental overdoses [11,50,51]. In clinical trials, the highest incidence of ADRs concerning nervous system disorders with a probable causal relationship to ibuprofen remained in the interval of 1–3%, even at high doses of 3200 mg per day. A lower frequency was reported for lower doses. Among the nervous system disorders, dizziness, headache, and nervousness were specifically mentioned, with the three reactions being usually considered non-serious [52]. On the other hand, ibuprofen recommendations were issued for doses higher than 2400 mg/day. No increase in risk was noted for doses up to 1200 mg/day [53].

The outcomes noted in our study for nervous system disorders were favorable for 46.8% of cases, while for psychiatric disorders, the opposite was noticed: the outcomes were unfavorable (especially for suicidal and self-injurious behaviors). The implications are serious and therefore careful attention should be given to mental health and side effects associated with common, highly consumed OTCs like ibuprofen. Moreover, through this study, we consider it important to raise awareness of the clinical context and possible risks associated with the general availability of ibuprofen as an OTC drug, especially in vulnerable groups such as teenagers or patients with a neuropsychiatric history.

In our study, ibuprofen presented a higher probability of reporting psychiatric and behavioral symptoms compared to ketoprofen, acetylsalicylic acid, and diclofenac. Similarly, but not emphasizing a particular NSAID, Jiang et al. presented five case reports detailing the relationship between common NSAIDs (e.g., diclofenac, ibuprofen, piroxicam) and the exacerbation of certain pre-existing psychiatric conditions, stating that NSAIDs should be prescribed with caution to patients with a history of depressive symptoms, suicide attempts, or any other paranoid psychiatric tendencies due to the risk of exacerbation of these episodes [54]. Compared to nimesulide, the present study showed a higher probability for reporting disturbances in thinking and perception. Contrary to this, a low risk of reporting a disturbance in thinking and perception could be noticed for ibuprofen compared to piroxicam.

The results of our study reveal that ibuprofen is associated more probably with depressed mood disorders compared to nimesulide, ketoprofen, and acetylsalicylic acid. Studies displayed controversial results regarding the association between depression and ibuprofen, highlighting the use of ibuprofen in the (complementary) treatment of depression [46,55,56]. Regarding anxiety, our study revealed that ibuprofen has a higher probability of reporting compared to ketoprofen, diclofenac, nimesulide, and acetylsalicylic acid, and a lower risk than other NSAIDs such as naproxen, meloxicam, celecoxib, and piroxicam.

According to our study, deliria in connection with ibuprofen was reported more frequently than for ketoprofen. Controversially, another study stated that ibuprofen and other NSAIDs may reduce the risk of postoperative delirium [57]. Furthermore, another study concluded that ibuprofen administered intraoperatively can significantly reduce the incidence of emergence agitation following general anesthesia with propofol and remifentanil in children [58].

The present findings also highlighted that personality disorders and disturbances in behavior are reported as more probable for ibuprofen than for ketoprofen, diclofenac, and etoricoxib. Similarly, Browning presented four patients with pre-existing psychiatric conditions that experienced exacerbation (paranoia, depression) when administered naproxen (six instances), diclofenac (two instances), and ibuprofen (one instance) [25]. These particular cases presented pre-existing psychiatric conditions as well as the lowest figures associated with ibuprofen (one instance).

In contrast to nimesulide, sleep disorders and disturbances are reported as more probable for ibuprofen. A study conducted by Roalsø et al. revealed a strong association of sleep disorders and anxiety with OTC analgesics (ibuprofen or similar, acetaminophen) [43,59]. A study including 37 subjects highlighted that ibuprofen also delayed the onset of the deeper stages of sleep through the suppression of melatonin levels [24]. Moreover, regarding nervous disorders, our study revealed that hypersomnia, narcolepsy, and sleep paralysis are reported more probably for ibuprofen than for acetylsalicylic acid (ROR: 4.42; 95% CI: 2.80–6.97), naproxen (ROR: 2.24; 95% CI: 1.17–4.29), and diclofenac (ROR: 2.20; 95% CI: 1.35–3.58). These findings could raise possible clinical implications that need further study.

Headaches were reported as more probable for ibuprofen than for acetylsalicylic acid, nimesulide, and ketoprofen, and by contrast as less probable than for naproxen, celecoxib, meloxicam, and diclofenac. These aspects might be explained by the fact that ibuprofen is a common OTC drug, used often as a first-line medication in mild to moderate pain management. Medication overuse headache is known to be associated with the long-term, frequent use of acute pain medication, NSAIDs included [23,60]. Our study revealed that movement disorders (including parkinsonism) and mental impairment disorders are reported as more probable for ibuprofen than for ketorolac and acetylsalicylic acid. However, some studies stated that the use of a non-aspirin NSAID, ibuprofen in particular, reduces the risk of Parkinson’s disease by 15% and, in contrast, the use of acetylsalicylic acid did not reveal any effect of this kind [23].

As shown by the results of our study, seizures (including subtypes) seemed to be reported as more probable for ibuprofen than for the majority of NSAIDs used in the current analysis [61]. However, recent data on the positive effects exerted by ibuprofen on brain function were reported [62]. Both positive and negative responses to ibuprofen use were also noticed during clinical trials [63] which can be attributed to dose dependance or the stage of the condition. As the BrainAGE study showed nearly identical positive responses for 200 mg and 600 mg of ibuprofen [41], further clinical trials are needed to clarify the influence of doses on ibuprofen effects, including ADRs.

### Strengths and Limitation of the Study

Continuous monitoring of suspected ADRs is considered essential to detect and to manage the emergence of new risks related to rare ADRs or those that occur with a delay or are not directly related to the pharmacological mechanism of the drug. Starting from these considerations, a strength of our study is represented by the analysis of real-world data, which includes a diverse population, such as elderly patients or patients with comorbidities and those taking concomitant medications, and a long time period. On the other hand, it is known that one of the general limitations of the spontaneous reporting system is underreporting, which can vary depending on both the drug and the ADRs. There are several reasons for underreporting or reporting bias, with the most important being a lack of awareness, difficulty in reporting, a perception that the reactions are minor, or a fear of consequences. Reporting in EudraVigilance does not necessarily include information regarding the medical or clinical history, comorbidities, concomitant medications, doses, time of use or indication. Thus, the aggregated data accessed does not allow for an assessment of the thorough association of ADRs and these factors, including possible drug–drug interactions. Moreover, for this type of pharmacovigilance study, an evaluation as such is not required. The quality of the reports submitted in EV could be affected, and some data may be missing or inaccurate. This is mainly due to the inhomogeneous reporting groups (e.g., healthcare professionals, pharmaceutical companies, and the general population). However, disproportionality analysis based on data obtained from a spontaneous reporting system is a validated method used in post-marketing surveillance of drug safety. Disproportionality analysis is essential for an improved comprehension of the real distribution of incidents and for the development of appropriate interventions.

## 4. Materials and Methods

### 4.1. Study Design

To evaluate neurologic and psychiatric disorders associated with ibuprofen use, a pharmacovigilance study was conducted. Data used for analysis was uploaded on the EV portal https://www.adrreports.eu/en/index.html (accessed on 3 June 2025) [32] until 1 June 2025. For the present study, aggregated data available on Level 1 (public access) have been analyzed. At this level, Individual Case Safety Reports (ICSRs) show data elements that are in compliance with the EU Data Protection legislation. Thus, open access is available for all academic researchers and the public, and no specific authorization is required for Level 1 [64]. in accordance with the Guideline on Good Pharmacovigilance Practices, marketing authorization holders are legally obligated to submit ICSRs to the EV portal for any serious ADRs occurring in non-EEA countries, provided that the medicinal product is authorized within the EEA [65]. Moreover, effective pharmacovigilance activity requires a proactive and systematic approach. Thus, patients’ safety increasingly depends on the integration of global safety data. Therefore, no exclusion criteria of cases related to the High-Level Term (HLT) of interest was applied in this study.

For the descriptive analysis, all ICSRs submitted for ibuprofen have been taken into consideration. Disproportionality analysis shows the probability of reporting ADRs by comparison with other drugs from the same therapeutic category [66]. For the present study, other NSAIDs from different chemical structure classes have been selected.

### 4.2. Material

Each ICSR includes general characteristics related to the patient (age, sex), origin, and reporter categories. There are several subcategories for age (not specified, 0–1 month, 2 months–2 years, 3–11 years, 12–17 years, 18–64 years, 65–85 years, and more than 85 years) and sex (not specified, male, female). The reports can originate from the EEA, or from non-EEA regions, and the reporter may be a healthcare professional or a non-healthcare professional. In order to facilitate international sharing of the regulatory information for medical products, rich and highly specific standardized medical terminology were developed in the late 1990s [67]. This collection was named the Medical Dictionary for Regulatory Activities (MedDRA) and included Preferred Terms (PTs) that can be used to report an ADR, and multiple PTs may be grouped under an HLT. Thus, a PT represents a “distinct descriptor (single medical concept) for a symptom, sign, disease diagnosis, therapeutic indication, investigation, surgical or medical procedure, and medical social or family history characteristic” [68].

PTs are also included in the 27 System Organ Classes (SOCs), which represent the highest level in the MedDRA hierarchy [69].

### 4.3. Descriptive and Disproportionality Analysis

The descriptive analysis was focused on 10 HLTs related to psychiatric conditions and 6 related to neurological conditions (Table S1 Supplementary Material). For each SOC (nervous system disorders and psychiatric disorders), the distribution of cases by age, sex, and reporter, as well the frequency of cases with unfavorable outcomes (fatal or not recovered/not resolved), was examined.

Disproportionality analysis is a statistical screening method used in pharmacovigilance to identify potential signals. This method compares the reporting frequency of a suspected ADR reported in databases for the studied drug by comparison with the frequency of the same ADR reported for other drugs. This method does not show a causal relationship between treatment and ADRs [70]. The probability of reporting ADRs from neurological and psychiatric areas was estimated based on the Reporting Odds Ratio (ROR) and a 95% confidence interval (CI). [70]. The ROR is a simple and widely used method in disproportionality analysis, valued for its ease of calculation and interpretation, as well as its applicability across diverse pharmacovigilance settings. According to the recommendations of the EMA, a signal could be considered disproportionated if a minimum 5 cases have been reported in the EV, and if the lower limit of a 95% CI is greater than 1 [71]. MedCalc application (Version 23.2.6) was used to calculate the ROR and a 95% CI [72].

$$ROR=\frac{a\times d}{b\times c}$$

where

*ROR* = Reporting Odds Ratio;

*a* = evaluated ADR for the targeted drug;

*b* = other ADRs for the targeted drug;

*c* = evaluated ADR for the drug used for comparison;

*d* = other ADRs for the drug used for comparison.

95% CI = exp (ln (ROR) − 1.96 × SE{ln(ROR)}) to exp (ln(ROR) + 1.96 × SE{ln(ROR)})

where

CI = confidence interval;

SE = standard error.

$$SE\{ln\left(ROR\right)\}=\sqrt{\frac{1}{a}+\frac{1}{b}+\frac{1}{c}+\frac{1}{d}}$$

### 4.4. Ethics

This study did not involve any human participants, and no personally identifiable information was collected or used. As a result, the research does not fall under the ethical guidelines that apply to studies involving human subjects. Therefore, review and approval by an ethics committee were not necessary [73].

## 5. Conclusions

The present study analyzed the spontaneous reports submitted for ibuprofen in the EV database in order to assess its neuropsychiatric safety profile. An important number of psychiatric (28.4%) and nervous system disorders (16.2%) have been reported with unfavorable outcomes. Compared to acetylsalicylic acid and ketoprofen (except for sleep disturbances, including subtypes), ibuprofen showed a higher probability of being reported in cases of neurologic and psychiatric disorders. In comparison with diclofenac, ibuprofen was more probable to be reported in cases of psychiatric disorders, except deliria, depressed mood disorders, and sleep disorders. Further, suicidal and self-injurious behaviors were reported with a higher likelihood in association with ibuprofen in comparison to all other NSAIDs. Though signals that correlate the use of ibuprofen and the probability of neuropsychiatric ADR reporting were detected, an established causality cannot be accurately determined. Considering that ibuprofen is frequently approved as an OTC drug and that it is available, in general, in retail outlets in many countries, its inappropriate use could lead to numerous side effects. Even though further research is needed to clarify the risks related to ibuprofen use, continued adverse event reporting (even when causality is uncertain) is important, contributes to the broader pharmacovigilance evidence base, and helps identify potential safety signals over time.

## Data Availability

The original contributions presented in this study are included in the article/supplementary material. Further inquiries can be directed to the corresponding author(s).

## Supplementary Materials

The following supporting information can be downloaded at https://www.mdpi.com/article/10.3390/ph18091301/s1, Figure S1: Distribution of cases by age within the Nervous System Disorders and Psychiatric Disorders System Organ Classes. Figure S2: Distribution of cases by reporter within the Nervous System Disorders and Psychiatric Disorders System Organ Classes. NS–not specified. Figure S3: Frequency of cases with unfavourable outcomes: fatal or not recovered/not resolved (NR/NRS). Table S1: High-Level Terms considered for the pharmacovigilance analysis. HLT–High Level Term; NEC-Not Elsewhere Classified; SOC–System Organ Class. Table S2: Reporting Odds Ratio (ROR, 95% CI) values for ibuprofen compared with other NSAIDs. CI–confidence interval; HLT–High Level Term; ROR -Reporting Odds Ratio; SOC–System Organ Class. Statistical significance is indicated if *p* < 0.05. A higher probability of reporting is considered when the lower bound of 95% CI is greater than 1.
