# Supplementary material for: Pharmacovigilance Insights into Ibuprofen’s Neuropsychiatric Safety: A Retrospective Analysis of EudraVigilance Reports
Source: Pharmaceuticals (Basel). 2025 Aug 29;18(9):1301. doi: 10.3390/ph18091301 (PMC12472944; doi:10.3390/ph18091301)
Supplement: Supplementary file 1 [file pharmaceuticals-18-01301-s001.zip › pharmaceuticals-3744045-supplementary.pdf]

Supplementary Material

Pharmacovigilance Insights into Ibuprofen's Neuropsychiatric Safety: A Retrospective Analysis of EudraVigilance Reports

Cristina Anamaria Buciuman, Carmen Maximiliana Dobrea, Anca Butuca, Adina Frum, Felicia Gabriela Gligor, Mihai O. Botea, Laura Grațiela Vicaș, Mariana Eugenia Mureșan, Octavia Gligor, Florin Maghiar, Alexia Manole and Claudiu Morgovan

Supplementary Figures

Figure S1. Distribution of cases by age within the Nervous System Disorders and Psychiatric Disorders System Organ Classes.

Figure S2. Distribution of cases by reporter within the Nervous System Disorders and Psychiatric Disorders System Organ Classes. NS – not specified.

Figure S3. Frequency of cases with unfavourable outcomes: fatal or not recovered/not resolved (NR/NRS).

Supplementary Tables

Table S1. High-Level Terms considered for the pharmacovigilance analysis. HLT – High Level Term; NEC - Not Elsewhere Classified; SOC – System Organ Class.

Table S2. Reporting Odds Ratio (ROR, 95% CI) values for ibuprofen compared with other NSAIDs. CI – confidence interval; HLT – High Level Term; ROR -Reporting Odds Ratio; SOC – System Organ Class. Statistical significance is indicated if $p < 0.05$. A higher probability of reporting is considered when the lower bound of 95% CI is greater than 1.

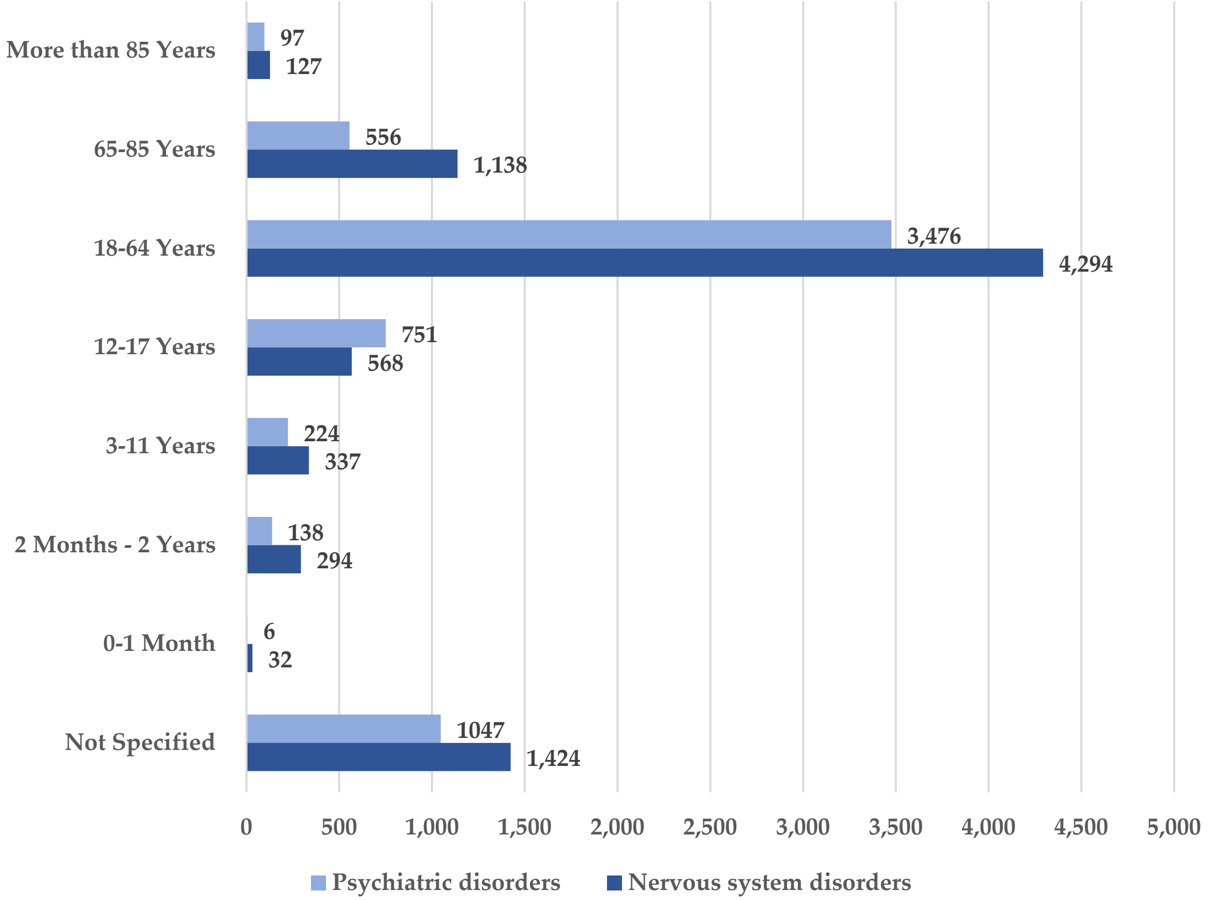

Figure S1. Distribution of cases by age within the Nervous System Disorders and Psychiatric Disorders System Organ Classes.

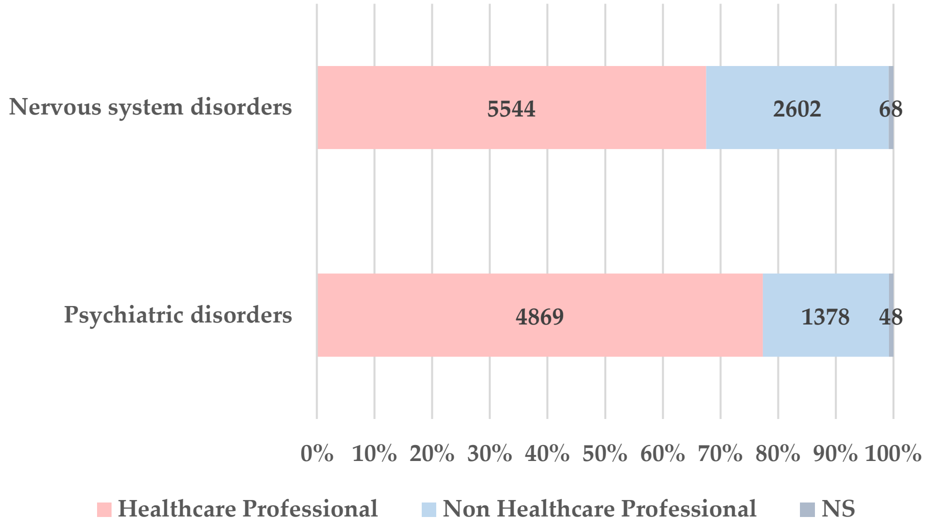

Figure S2. Distribution of cases by reporter within the Nervous System Disorders and Psychiatric Disorders System Organ Classes. NS – not specified.

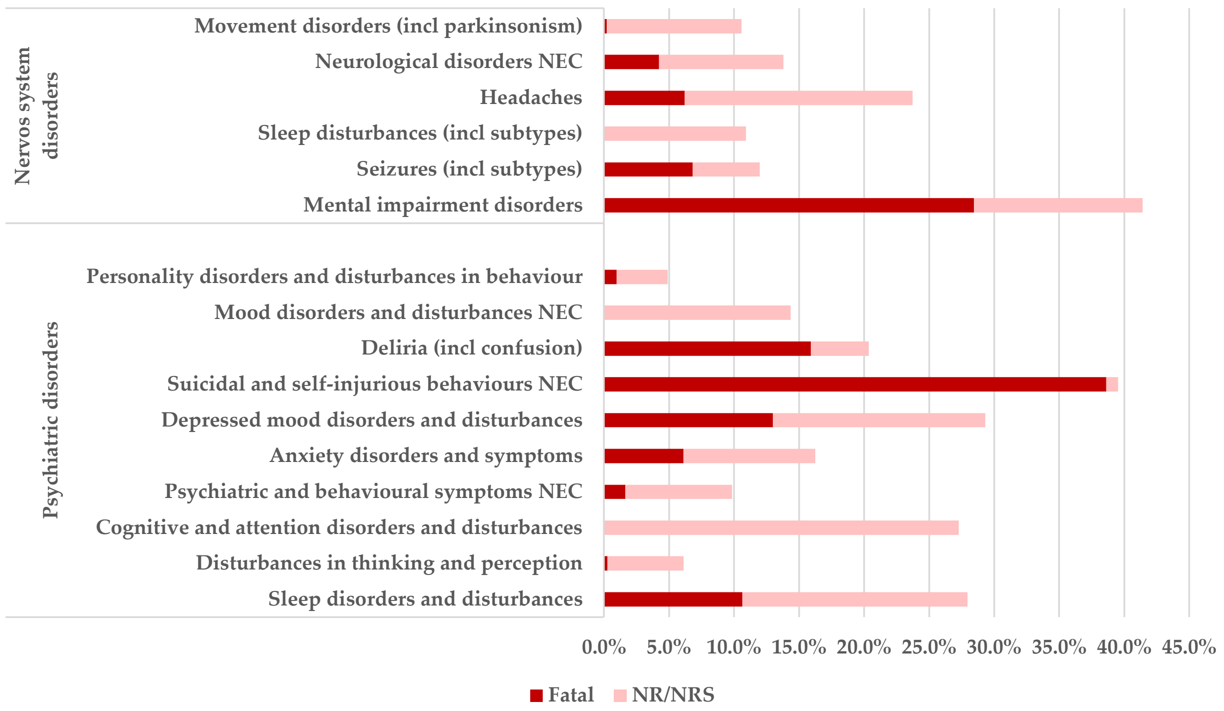

Figure S3. Frequency of cases with unfavourable outcomes: fatal or not recovered/not resolved (NR/NRS).

Table S1. High-Level Terms considered for the pharmacovigilance analysis. HLT – High Level Term; NEC - Not Elsewhere Classified; SOC – System Organ Class.

SOC	HLT
**Psychiatric disorders**	Sleep disorders and disturbances
	Disturbances in thinking and perception
	Cognitive and attention disorders and disturbances
	Psychiatric and behavioural symptoms NEC
	Anxiety disorders and symptoms
	Depressed mood disorders and disturbances
	Suicidal and self-injurious behaviours NEC
	Deliria (including confusion)
	Mood disorders and disturbances NEC
	Personality disorders and disturbances in behaviour
**Nervous system disorders**	Sleep disturbances (including subtypes)
	Headaches
	Neurological disorders NEC
	Movement disorders (including parkinsonism)
	Mental impairment disorders
	Seizures (including subtypes)

Table S2. Reporting Odds Ratio (ROR, 95% CI) values for ibuprofen compared with other NSAIDs. CI – confidence interval; HLT – High Level Term; ROR -Reporting Odds Ratio; SOC – System Organ Class. Statistical significance is indicated if $p < 0.05$. A higher probability of reporting is considered when the lower bound of 95% CI is greater than 1.

SOC	HLT	Drug used for comparison	ROR	95% CI lower bound	95% CI upper bound	P value
	**Psychiatric and behavioural symptoms NEC**	Meloxicam	0.93	0.40	2.15	P = 0.8612
		Naproxen	0.94	0.61	1.47	P = 0.7977
		Celecoxib	1.73	0.98	3.05	P = 0.0565
		Etoricoxib	1.86	0.80	4.31	P = 0.1465
		Diclofenac	2.34	1.46	3.75	P = 0.0004
		Acetylsalicylic acid	2.75	1.89	3.99	P < 0.0001
		Ketoprofen	3.21	1.29	7.98	P = 0.0123
	**Cognitive and attention disorders and disturbances**	Acid acetilsalicilic	0.85	0.43	1.71	P = 0.6538
		Diclofenac	2.02	0.70	5.82	P = 0.1917
	**Disturbances in thinking and perception**	Piroxicam	0.52	0.37	0.72	P = 0.0001
		Etoricoxib	0.69	0.55	0.86	P = 0.0009
		Naproxen	0.74	0.62	0.87	P = 0.0003
		Ketorolac	0.84	0.58	1.20	P = 0.3379
		Celecoxib	1.22	1.00	1.49	P = 0.0469
		Meloxicam	1.38	0.92	2.06	P = 0.1239
		Diclofenac	1.70	1.43	2.01	P < 0.0001
		Ketoprofen	2.75	1.96	3.88	P < 0.0001
		Acetylsalicylic acid	4.47	3.76	5.33	P < 0.0001
		Nimesulide	4.60	2.18	9.73	P = 0.0001
	Sleep disorders and disturbances	Naproxen	0.31	0.28	0.34	P < 0.0001
		Celecoxib	0.33	0.30	0.36	P < 0.0001
		Meloxicam	0.43	0.37	0.52	P < 0.0001
		Diclofenac	0.53	0.49	0.58	P < 0.0001
		Piroxicam	0.54	0.42	0.69	P < 0.0001
		Ketorolac	0.67	0.54	0.85	P = 0.0008
		Etoricoxib	0.76	0.64	0.89	P = 0.0006

		Ketoprofen	2.49	1.98	3.12	P < 0.0001
		Acetylsalicylic acid	2.50	2.26	2.77	P < 0.0001
		Nimesulide	3.73	2.34	5.96	P < 0.0001
	**Anxiety disorders and symptoms**	Naproxen	0.61	0.55	0.68	P < 0.0001
		Meloxicam	0.61	0.51	0.74	P < 0.0001
		Celecoxib	0.68	0.61	0.76	P < 0.0001
		Piroxicam	0.72	0.55	0.92	P = 0.0098
		Etoricoxib	0.99	0.84	1.17	P = 0.9334
		Ketorolac	1.07	0.83	1.39	P = 0.5911
		Diclofenac	1.27	1.15	1.40	P < 0.0001
		Nimesulide	2.02	1.47	2.79	P < 0.0001
		Acetylsalicylic acid	2.45	2.23	2.70	P < 0.0001
		Ketoprofen	2.93	2.34	3.67	P < 0.0001
	**Depressed mood disorders and disturbances**	Celecoxib	0.34	0.30	0.39	P < 0.0001
		Naproxen	0.38	0.33	0.43	P < 0.0001
		Meloxicam	0.51	0.40	0.65	P < 0.0001
		Piroxicam	0.56	0.40	0.78	P = 0.0006
		Diclofenac	0.75	0.66	0.86	P < 0.0001
		Etoricoxib	1.07	0.83	1.38	P = 0.5851
		Ketorolac	1.42	0.91	2.20	P = 0.1201
		Acetylsalicylic acid	1.56	1.38	1.77	P < 0.0001
		Ketoprofen	2.89	2.07	4.03	P < 0.0001
		Nimesulide	5.10	2.41	10.77	P < 0.0001
	**Suicidal and self-injurious behaviours NEC**	Meloxicam	1.49	1.27	1.75	P < 0.0001
		Naproxen	1.59	1.47	1.73	P < 0.0001
		Acetylsalicylic acid	3.10	2.92	3.29	P < 0.0001
		Ketorolac	3.20	2.47	4.14	P < 0.0001
		Piroxicam	4.31	3.03	6.12	P < 0.0001
		Ketoprofen	4.86	4.11	5.75	P < 0.0001
		Diclofenac	4.87	4.42	5.35	P < 0.0001
		Nimesulide	4.91	3.67	6.56	P < 0.0001
		Celecoxib	7.22	6.15	8.48	P < 0.0001
		Etoricoxib	9.95	7.52	13.17	P < 0.0001
	**Deliria (incl confusion)**	Naproxen	0.41	0.37	0.46	P < 0.0001

		Piroxicam	0.43	0.34	0.55	P < 0.0001
		Celecoxib	0.46	0.41	0.51	P < 0.0001
		Meloxicam	0.46	0.38	0.56	P < 0.0001
		Diclofenac	0.54	0.49	0.60	P < 0.0001
		Etoricoxib	0.89	0.74	1.07	P = 0.2288
		Ketorolac	1.02	0.76	1.38	P = 0.8842
		Nimesulide	1.31	0.96	1.78	P = 0.0857
		Acetylsalicylic acid	1.48	1.34	1.63	P < 0.0001
		Ketoprofen	2.00	1.60	2.49	P < 0.0001
	**Personality disorders and disturbances in behaviour**	Ketorolac	1.04	0.48	2.24	P = 0.9198
		Naproxen	1.31	0.89	1.92	P = 0.1705
		Meloxicam	1.33	0.62	2.86	P = 0.4651
		Celecoxib	1.61	1.05	2.46	P = 0.0278
		Etoricoxib	2.08	1.05	4.11	P = 0.0357
		Diclofenac	2.61	1.78	3.81	P < 0.0001
		Acetylsalicylic acid	2.61	1.96	3.47	P < 0.0001
		Ketoprofen	2.68	1.40	5.13	P = 0.0029
	**Mood disorders and disturbances NEC**	Meloxicam	0.58	0.41	0.83	P = 0.0023
		Naproxen	1.04	0.83	1.32	P = 0.7241
		Celecoxib	1.20	0.94	1.54	P = 0.1469
		Ketorolac	1.54	0.84	2.82	P = 0.1621
		Diclofenac	1.69	1.36	2.10	P < 0.0001
		Piroxicam	1.73	0.82	3.67	P = 0.1534
		Etoricoxib	2.29	1.43	3.65	P = 0.0005
		Nimesulide	3.38	1.50	7.60	P = 0.0032
		Ketoprofen	3.47	2.15	5.60	P < 0.0001
		Acetylsalicylic acid	3.49	2.85	4.28	P < 0.0001
**Nervous system disorders**	**Sleep disturbances (incl subtypes)**	Ketorolac	0.78	0.31	1.96	P = 0.6044
		Meloxicam	0.84	0.36	1.94	P = 0.6782
		Etoricoxib	0.92	0.48	1.75	P = 0.7881
		Celecoxib	1.23	0.73	2.08	P = 0.4309
		Diclofenac	2.20	1.35	3.58	P = 0.0015
		Naproxen	2.24	1.17	4.29	P = 0.0144
		Acetylsalicylic acid	4.42	2.80	6.97	P < 0.0001

	Headaches	Naproxen	0.55	0.51	0.59	P < 0.0001
		Celecoxib	0.73	0.68	0.79	P < 0.0001
		Meloxicam	0.78	0.68	0.90	P = 0.0008
		Diclofenac	0.86	0.81	0.92	P < 0.0001
		Piroxicam	0.97	0.78	1.19	P = 0.7400
		Ketorolac	1.02	0.85	1.22	P = 0.8508
		Etoricoxib	1.04	0.92	1.17	P = 0.5607
		Ketoprofen	1.63	1.44	1.84	P < 0.0001
		Nimesulide	2.07	1.64	2.60	P < 0.0001
		Acetylsalicylic acid	2.17	2.03	2.31	P < 0.0001
	Neurological disorders NEC	Naproxen	0.55	0.53	0.58	P < 0.0001
		Celecoxib	0.70	0.67	0.74	P < 0.0001
		Meloxicam	0.76	0.69	0.83	P < 0.0001
		Etoricoxib	0.76	0.71	0.82	P < 0.0001
		Diclofenac	0.81	0.78	0.85	P < 0.0001
		Piroxicam	1.03	0.90	1.18	P = 0.6977
		Ketorolac	1.12	0.99	1.26	P = 0.0740
		Acetylsalicylic acid	1.33	1.28	1.38	P < 0.0001
		Ketoprofen	1.92	1.77	2.08	P < 0.0001
		Nimesulide	2.81	2.38	3.31	P < 0.0001
	Movement disorders (incl parkinsonism)	Ketorolac	0.55	0.42	0.72	P < 0.0001
		Meloxicam	0.90	0.67	1.21	P = 0.4790
		Naproxen	0.92	0.79	1.08	P = 0.3068
		Celecoxib	0.97	0.82	1.14	P = 0.7149
		Piroxicam	1.07	0.70	1.63	P = 0.7463
		Diclofenac	1.11	0.97	1.27	P = 0.1218
		Etoricoxib	1.16	0.91	1.48	P = 0.2188
		Nimesulide	1.25	0.88	1.78	P = 0.2156
		Ketoprofen	1.61	1.27	2.04	P = 0.0001
		Acetylsalicylic acid	2.41	2.12	2.74	P < 0.0001
	Mental impairment disorders	Naproxen	0.19	0.17	0.22	P < 0.0001
		Piroxicam	0.21	0.17	0.27	P < 0.0001
		Celecoxib	0.25	0.22	0.29	P < 0.0001
		Meloxicam	0.34	0.27	0.43	P < 0.0001

	Diclofenac	0.46	0.40	0.52	P < 0.0001
	Etoricoxib	0.97	0.75	1.26	P = 0.8170
	Ketorolac	0.97	0.65	1.45	P = 0.8989
	Acetylsalicylic acid	1.94	1.68	2.24	P < 0.0001
	Ketoprofen	3.60	2.42	5.36	P < 0.0001
**Seizures (incl subtypes)**	Ketorolac	0.54	0.41	0.71	P < 0.0001
	Meloxicam	0.88	0.65	1.21	P = 0.4332
	Diclofenac	0.95	0.83	1.09	P = 0.4379
	Celecoxib	1.30	1.07	1.57	P = 0.0075
	Naproxen	1.30	1.08	1.57	P = 0.0059
	Piroxicam	1.82	1.02	3.23	P = 0.0418
	Acetylsalicylic acid	2.26	1.98	2.59	P < 0.0001
	Nimesulide	2.81	1.62	4.88	P = 0.0002
	Etoricoxib	3.27	2.16	4.93	P < 0.0001
	Ketoprofen	16.09	7.62	33.96	P < 0.0001
